# Consumer Interaction with Sustainability Labelling on Food Products: A Narrative Literature Review

**DOI:** 10.3390/nu15173837

**Published:** 2023-09-02

**Authors:** Brian Cook, João Costa Leite, Mike Rayner, Sandro Stoffel, Elaine van Rijn, Jan Wollgast

**Affiliations:** 1 Independent Researcher, Oxford OX4 3UD, UK; brian@foodsystemresearch.com; 2 European Commission, Joint Research Centre (JRC), 21027 Ispra, Italy; joao.leite@ec.europa.eu (J.C.L.); elaine.van-rijn@ec.europa.eu (E.v.R.); 3 Nuffield Department of Population Health, University of Oxford, Oxford OX3 7LF, UK; mike.rayner@ndph.ox.ac.uk; 4 Research Department of Behavioural Science and Health, University College London (UCL), London WC1E 6BT, UK; sandro.stoffel@unibas.ch; 5 Institute of Pharmaceutical Medicine (ECPM), University of Basel, CH-4056 Basel, Switzerland

**Keywords:** food labelling, sustainability labelling, consumer behaviour, effectiveness, review

## Abstract

Sustainability labelling on food products can help consumers make informed purchasing decisions and support the urgent transition to sustainable food systems. While there is a relatively robust body of evidence on health and nutrition labelling, less is known about the effectiveness of sustainability labelling in facilitating sustainable food choices. This paper investigates the impact of sustainability labelling on consumer understanding, attitudes, and behaviour to support a more nuanced, detailed, and holistic understanding of the evidence. Using a narrative literature review methodology, the paper assesses studies covering environmental, social, and/or animal welfare aspects of sustainability labelling on food products. We found that consumer understanding of sustainability information is often limited, which could hinder behaviour change. While sustainability labelling can influence consumer attitudes and purchasing behaviours, evidence from real consumer settings tends to show small effect sizes. Consumers are generally willing to pay more for sustainability-labelled products, and organic labelling often leads to the highest reported willingness to pay. The review emphasises the importance of trust, suggesting a preference for labelling backed by governments or public authorities. Sustainability labelling that uses intuitively understandable cues has an increased impact, with visual aids such as traffic light colours showing promise. We conclude that further research is needed in real-world settings, using representative populations and exploring the influence of demographic factors, values, and attitudes.

## 1. Introduction

The urgent need to transition toward sustainable food systems is becoming more salient than ever, given the substantial contribution of the food systems to sustainable development. The effects of the current consumption patterns pose an imminent threat to public health, environment, and international climate objectives. Achieving healthy, sustainable diets requires food environments that promote transparency and better information to consumers, enabling them to make sustainable food choices [1]. One potential strategy to shift consumer behaviour is the use of sustainability labelling on food and drink products. This can provide consumers with an understanding of the environmental, social, and animal welfare implications associated with their choices, potentially leading to more sustainable food purchases. The labelling could also serve as a catalyst for supply chain actors to improve their sustainability performance. Currently, a diverse range of sustainability labels is present in the European market and globally. A recent mapping study found that nearly 50% of new food product launches include a sustainability claim, with about 20% of them providing sustainability information through logos [2].

While there is a relatively robust body of evidence on health and nutrition labelling [3], the same is not true for sustainability labelling. With the urgency of environmental concerns, a clearer understanding of the research on this topic to date, its findings and gaps is needed. Evidence from diverse studies suggests that sustainability labelling can influence consumer behaviour [4,5]. However, the studies are heterogeneous, and the literature has not been analysed from a broad framing of sustainability to identify insights and research gaps.

The definition of labelling for this paper is derived from European Union (EU) regulations which refer to labelling as “any words, particulars, trademarks, brand name, pictorial matter or symbol relating to a food and placed on any packaging, document, notice, label, ring or collar accompanying or referring to such food” [6]. This can include ingredients, usage instructions, nutrition information, or, relevant for the purpose of this review, information or claims about sustainability aspects related to the product. A label, on which labelling appears, is defined as “any tag, brand, mark, pictorial or other descriptive matter, written, printed, stenciled, marked, embossed or impressed on, or attached to the packaging or container of food” [6]. Sustainability labelling can include neutral information about the food, its production processes, or the packaging and/or claims about the sustainability performance of those. While the terminology used in this paper, such as labelling, is derived from the EU, the scope of this review remains global in nature.

**Figure 1:**
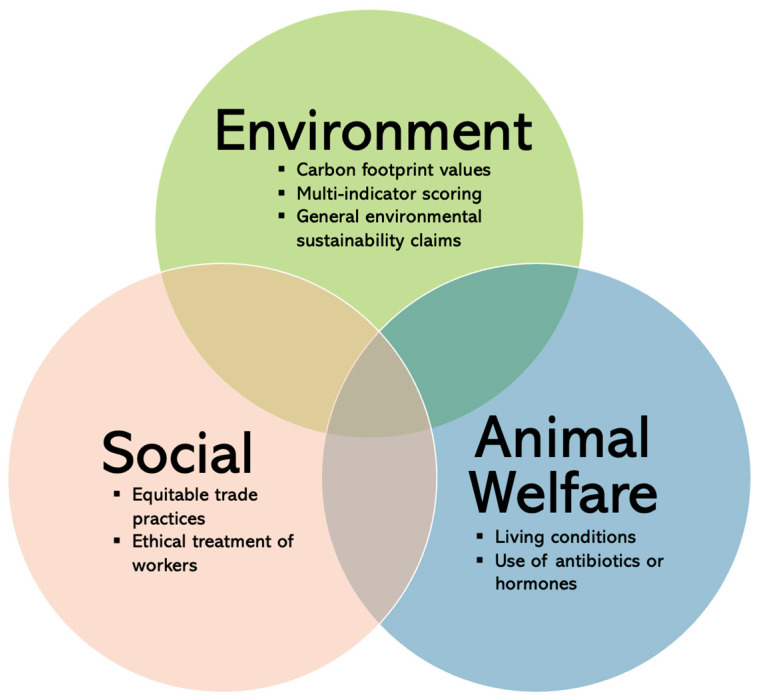
Types of Existing Sustainability Labelling by Objective.

This review looks at sustainability from the perspective of environmental (e.g., carbon footprint, biodiversity loss), social (e.g., fair trade, equal treatment of all workers), and animal welfare (e.g., living conditions, use of antibiotics or hormones) objectives (see Figure 1) [7]. Different types of labelling can encompass one or more sustainability dimensions and different aspects of food production (e.g., Fairtrade focuses on social sustainability but also has some environmental objectives; the EU organic label covers environmental and animal welfare standards) [8]. Each of the three sustainability objectives also overlaps with the broad concept of health. The link between health and environmental sustainability reflects the holistic relationship between humans and their natural surroundings. Climate change, in particular, can have direct health impacts. For example, extreme weather events can lead to a rise in infectious diseases, as well as respiratory and cardiovascular outcomes [9]. Social sustainability indirectly supports individual health by safeguarding fair socio-economic conditions for workers and promoting ethical trade practices. Animal welfare labelling relates to the respect for animal lives and is linked to human health through, for example, preventing the spread of zoonotic diseases and reducing the risk of foodborne illnesses. While there is a clear connection to human health, this review specifically focuses on the three dimensions of sustainability labelling, excluding any research on health-only aspects such as nutrition labelling, which has been covered extensively elsewhere [10,11].

The principal goal of this review is to consolidate and critically assess the evidence surrounding sustainability labelling, investigating its influence on consumer understanding, attitudes, and behaviour. By delving into the current state of research in this field, this review aims to clarify the impact of sustainability labelling in promoting more sustainable food choices to support a more nuanced, detailed, and holistic understanding of the evidence. Enhancing understanding in this area is crucial, as it would not only broaden our comprehension of consumer behaviour but also provide us with the tools to inspire change through policy making and future research agendas.

## 2. Materials and Methods

### Scope of the Review

A narrative literature review was conducted using searches of the Cochrane Central Database of Controlled Trials, the Cochrane Database of Systematic Reviews, Embase, CAB Abstracts, Ovid MEDLINE(R), PsycINFO, and the Science Citation Index in July 2022. The searches identified original research related to sustainability labelling on food published since 2000. Keywords used in the searches included: ecolabel/eco-label$, environment$, ecolog$, sustain$, green$, label, carbon footprint, recycl$, organic$, animal welfare, social sustainability, food preferences, eating attitudes, consume/consumption, eat$, diet$, food, drink$, behavio?r$, choice$, decision$, select$, purchas$, buy$, sale$.

Studies were included if they focused on any aspects of consumer attitudes (including expectations, awareness, perceptions, preferences, or understanding), behaviour (product selection, purchase, and/or consumption), or other consumer interactions with sustainability labelling (including consumer trust) on food, including indicators of environmental, social, or animal welfare impacts. There were no restrictions by geographic focus, population, or study design. Peer-reviewed papers were excluded if they were written in a language other than English, but grey literature searches included any language. The initial searches yielded 1018 papers, which were reduced to 635 after removing duplicates. After title and abstract screening for relevance, 167 relevant papers were identified for this review, which included papers with a sole focus on one aspect of sustainability labelling and those focused on multiple aspects. The majority focused on environmental impact labelling (including “ecolabels”, “eco-labels”, and carbon footprint) (*n* = 101). A substantial number of studies assessed the impacts of organic labelling on food products (*n* = 58), while far fewer explored social sustainability (*n* = 11) or animal welfare labelling (*n* = 5). The searches also uncovered 9 systematic reviews that dealt with different aspects of sustainability labelling.

To expand the scope of the review, online searches of grey literature, sourced primarily from government, industry, and organisation publications, were conducted. In a further step, to address the publication bias in academic research whereby studies with null results are less likely to be written up [12], the research team developed a short online questionnaire and distributed it among 192 European sustainability-related label owners and third-party certification agencies, identified in a previous mapping study of sustainability-related labels in food products [2]. The questionnaire asked about types of unpublished consumer research related to sustainability food labelling that organisations administer or manage. The questionnaire was distributed through email and online contact forms. The team sent three reminders to the organisations, and 30 questionnaires were completed. Fifteen respondents reported having evaluated their labelling, and seven agreed to share documents related to the findings. All documents, regardless of language, were reviewed, as were other non-peer-reviewed publications found in targeted grey literature web searches. Information from these publications is cited in the report where relevant. Detailed results of the labelling questionnaire can be found in Appendix A.

## 3. Results

**Figure 2:**
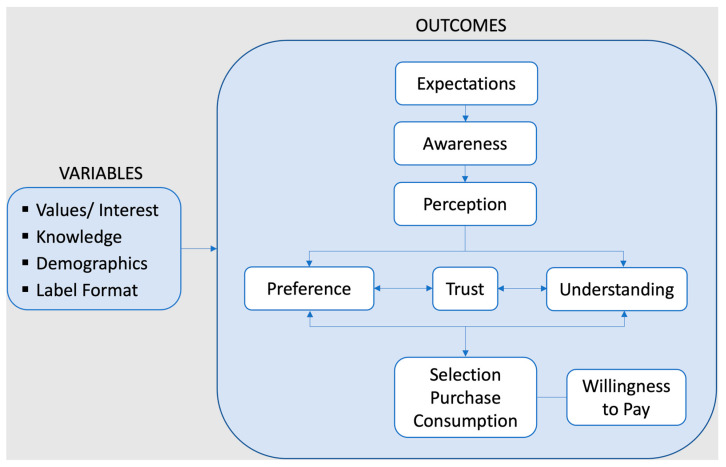
Consumer Interaction with Sustainability Labelling. Adapted from Grunert, K. G., & Wills, J. M. (2007) [13].

To organize the results of the review, Figure 2 proposes a framework covering variables and outcomes related to consumer attitudes and behaviour relevant to sustainability labelling. The framework is adapted from Grunert and Wills (2007), who drew on theories of consumer decision making and attitude formation and change [13]. Consumer decision-making research is core to the study of sustainability labelling as it speaks to how choices are made among competing products and the role that information plays. However, consumer decisions are not separate isolated events. As shown by research on attitude formation and change, when people encounter product information, they form attitudes, evaluate its meaning, and judge whether it is aligned with their values and interests, all of which precedes its effect on their behaviour [14].

Figure 2 proposes a schematic depiction of outcomes on which different types of sustainability labelling can have an influence. Consumer expectations of labelling information and claims can precede their exposure to them and help form the basis upon which a label or its accompanying product’s performance is judged during use [15]. Awareness will also be investigated in this review, as only labels to which consumers are exposed can have an effect. Once aware of sustainability labelling and exposed to them, such as on a supermarket shelf, consumer perception follows, which can occur consciously or unconsciously. Once a consumer takes in the visual and informational cues of the label, this leads to understanding which is the meaning that is attached to the label. This understanding may or may not align with what the label owners intended. When the labelling is perceived and its information is processed, this may lead to consumer preference for, or liking of, the label, and possibly also the labelled product. Consumers can understand sustainability labelling correctly and not like it, and vice versa. How consumers interpret labelling determines whether they trust the information and claims being conveyed. In the absence of trust, people will not have belief in the credibility or accuracy of the labelling and are unlikely to be influenced by it. Lastly, the labelling can lead to consumers selecting, purchasing, and consuming the labelled food. A related purchasing outcome is consumers’ willingness to pay a premium for sustainably-labelled food products, which will also be assessed in this review. Drawing on research from nutrition labelling [13,16], it is reasonable to expect that similar predictors might play a role in the effects of sustainability labelling, including consumer interest in, or knowledge of, sustainability concerns, different demographic variables, and the format of the labelling itself. The review will analyze evidence on the role of these predictors of consumer behaviour related to sustainability labelling. It should also be noted that sustainability labelling is likely to interact with other types of product labelling (marketing claims, health information and claims, ingredients, food safety information, etc.). However, this has only been explored in a limited number of studies [17,18], which we have referenced.

### 3.1. Expectations, Awareness, and Perception

Consumer expectations are not well studied or defined in the literature. Expectations about food labelling, for the purpose of this review, refer to what consumers want, hope, or need to see in labelling information, claims, and formats. It can also refer to the “feeling or belief” about the meaning or value. In this way, expectations are closely linked to perceptions and preferences.

Among EU consumers, there appears to be a strong interest in sustainability labelling. A 2020 survey conducted in the 27 EU Member States found that a large majority of consumers (88%) agreed that sustainability information should be made mandatory on food labels. A June 2022 survey among United Kingdom (UK) consumers found similar results, showing a strong interest in sustainability labelling (81%) but far fewer people believing that labelling is the most important tool to support more sustainable consumer choices. Less than a third of the respondents (32%) said that “better carbon footprint labelling” was what they needed to adopt a more sustainable lifestyle. Other research has looked at the information and presentation that consumers want on food labels. Most of these insights derive from qualitative research, often surveys and focus groups, which offer valuable insights about perceptions and intentions but not about actual food purchasing behaviour. For example, traffic light colours are widely considered to be well understood and have a greater impact versus black and white labels [19,20]. This aligns with nutrition front-of-package labelling research showing that consumers generally prefer simple, colour-coded labelling that are more easily understood to more complex, monochrome labels [3].

There is some evidence that particular consumer groups find sustainability labelling more appealing and acceptable compared to others. However, the links are not consistent across studies. Potter et al. (2021) systematically reviewed 56 studies and found modest evidence that, compared to the average study sample effect, environmental sustainability labelling influenced women, in particular, those of higher income or education, but the effects of age were mixed [4]. The effect of gender may stem from women having a higher concern for environmental issues but could also be a sampling bias, given that many studies recruit those who are responsible for household food purchasing decisions, who tend to be disproportionally female. A review of consumer perceptions of carbon footprint labelling (showing only a food’s linked emissions) was firmer in its conclusions that females and those with higher income and educational levels reported a more positive attitude toward the labels [21]. Another systematic review found that higher levels of education and the female gender played significant roles in increasing the impact of environmental impact labelling, while a consumer focus on low prices reduced the impact [18]. Income differences were found to have effects on the level of understanding of carbon footprint labelling among consumers from the UK, France, Germany, Spain, Sweden, and Poland [22]. Values also play a role in driving the appeal of sustainability labelling among consumers. Those with greater concern for environmental sustainability report valuing it more compared to other product attributes and feeling greater trust in the label [20]. There may also be a perceived benefit of being viewed by one’s peers as a “green” consumer that sustainability labelling offers [23].

Many studies on organic labelling found significant increases in consumer preferences and purchasing behaviour. A systematic review of labelling interventions found that consumers showed a strong preference for organic labels [18]. People often cited health as their primary motivation for purchasing these products. In a separate systematic review on sustainability labels, Potter et al. (2021) included 25 studies examining the impact of organic labelling and found that almost all showed an increase in consumer purchases of organic products. This included 4 out of 4 studies using an organic logo, 10 of 11 studies testing a text-only organic label, and 8 of 10 studies that employed a combined text and logo organic label. Among the studies that tested a hypothetical selection of foods, two tested organic labelling, and both found an effect compared to the control (no label). Three organic labelling studies tested the effect on real-world purchases, and all found an increase in consumer buying behaviour for these products.

Organic standards overlap with animal welfare rules, and research shows that consumers correctly interpret organic to mean animals are treated according to higher welfare standards compared to conventional animal agriculture [24]. In one discrete choice experiment, the participants did not show an increased preference for animal welfare-labelled products when accompanied by an organic claim, as, presumably, they perceived organic to already cover these standards. But participants did report increased preference for animal welfare labelling when it was combined with local food labelling, as these two claims were seen as separate [25]. The authors did not report the strength of the effect, only the significance of the interaction between the labels.

A meta-analysis of 53 consumer studies on animal welfare labelling found strong evidence that “considerable shares of consumers” have a positive attitude toward animal welfare-friendly husbandry systems and are willing to pay a price premium for these products [26]. A survey conducted across all EU countries found that the majority of consumers would like more information on animal welfare conditions to make informed decisions [27]. Consumers in northern and western EU Member States showed a slightly higher level of awareness, but demand for information on animal welfare was fairly consistent across the EU. Younger people and females reported the highest interest in the implementation of an animal welfare labelling scheme.

Research on the effect of Fairtrade labelling has mostly focused on consumer perceptions and preferences, with few studies on its effects on actual purchase or consumption. A study of 179 Belgian consumers had them taste three pairs of food items (nuts, juice, and chocolate), with one product in each pair labelled as “conventional”, and the other labelled as “Fairtrade”. Each product in the pair was actually a Fairtrade item. The Fairtrade-labelled product was associated with higher preference scores [28]. A higher level of preference for Fairtrade versus conventional foods was also found in a German study on coffee and chocolate [29] and in China, where Fairtrade labelling resulted in a greater preference for tea among undergraduate students [30]. Other studies found a preference for the Fairtrade label, but only when people had a pre-existing positive attitude toward Fairtrade values [31,32].

### 3.2. Preference and Understanding

While an apparent increase in consumer support for sustainability labelling is encouraging, it is also important to know whether people understand these labels, whether they understand them as intended, and if not, what the sources of confusion are. While labelling can influence consumers even when they do not fully understand it, for example, when companies leverage emotional appeals to sell a product, consumers say that they want more information about the sustainability impacts of products and for companies to be more transparent [33]. Food labelling can be misinterpreted, as well as intentionally distorted to mislead consumers. EU consumers are wary of potential “greenwashing”, with some believing that companies intentionally create complex labelling that the average consumer is unable to understand [33].

Research has documented the extent to which consumers misinterpret existing sustainability information and claims on food products. Several studies have focused on carbon footprint labelling on food that provide some measure of the greenhouse gas emissions linked to the product. The research points to consumers generally having a poor understanding of carbon footprint labelling that provides specific numerical values in the absence of other, more intuitive, visual or informational cues. Too much information can cause an information overload, leading some consumers to disregard the label [34]. People report finding it difficult to understand the meaning of greenhouse gas emission values for their personal context [20]. Some consumers misinterpret carbon footprint labelling as referring to the pollution or contamination in a food, rather than to the emissions linked to its production [35]. When the labelling was redesigned using intuitive symbols or traffic light colours, consumer understanding was shown to increase [21]. Intuitive warning labels, such as those with an octagonal shape, may also be worth investigating. Two recent experiments in virtual settings that tested warning labels on red meat found no significant effect of environmental warnings, but health warning messages were perceived to be effective by consumers [36,37].

A “health halo” effect can be found in several studies on organic labels, revealing a common consumer perception that more environmentally sustainable products offer health benefits [18]. For example, in a survey testing preferences for health and organic claims for yogurt, the participants perceived organic yogurt to have a higher health score than yogurt with specific health claims [38]. Dangour et al. (2010) found the “health halo” to be especially prominent for organic-labelled foods, which many consumers believed to be a healthier alternative to conventional products, despite a lack of convincing evidence to support their nutritional superiority [39]. Given the strong health perception of organic products among consumers, there can be a concern that marketers could exaggerate the benefits of organic certification.

### 3.3. Trust

Trust is an essential component of sustainability labelling as, in its absence, labelling will have little or no impact on consumer behaviour [40]. In today’s complex global food system, some consumers find it difficult to trust food labelling and sources of information on food’s health, social, and environmental impacts. Mistrust in food system actors presents a problem for the food industry and for governments in attempting to shift consumer habits toward healthier and more sustainable choices. Consumers are constantly forming judgements about whether to trust specific products and food system actors when they navigate shopping environments.

Several studies explored factors influencing consumer trust in sustainability labels. The messenger behind labelling information or claim has been found to be a key factor. The industry has spearheaded many efforts to promote food and non-food products as better for the environment. However, sustainability terms such as “green” that are not backed by verified claims have been overused by companies, and this can reduce the credibility of other sustainability labels. The proliferation of sustainability schemes has caused consumer confusion and scepticism about the alleged sustainability claims [41].

Tonkin et al. (2015) reported mixed findings from a systematic review of 27 studies on who is most trusted to deliver food labelling information or claims (including nutrition, social, and environmental) [40]. This level of trust can vary depending on regional differences in institutional trust and cultural norms. A 2021 survey among 20,000 EU consumers in 18 Member States showed relatively high levels of trust in EU authorities in terms of their ability to administer sustainability standards [33]. A survey of EU consumers in all Member States showed that an animal welfare labelling scheme backed by non-governmental organisations (NGOs) and EU public authorities would engender greater trust compared to one managed by food companies [27]. Consumers in Denmark were found to have high levels of trust in organic labelling compared to those in the UK, Sweden, and the U.S.A., even after controlling for generalised institutional and social trust. The Danish system is very much led by the government, leading the authors to conclude that significant state involvement increases consumer trust in organic labelling [42]. Studies from North America found similarly high levels of trust in organic certification, with some suggesting that the United States Department of Agriculture (USDA) organic logo is trusted more than generic organic logos [43]. However, Nagy et al. (2022) also found that consumers from developing countries are often sceptical of certifications from their own country.

Trust in sustainability certification claims has been linked to the credibility of the overseeing organisation [44]. Institutional trust plays a large role in promoting a belief in the credibility of sustainability labelling. An analysis of consumer responses in France, Germany, and Serbia found that increased knowledge of the third-party certification, supported by communication campaigns, increased trust in these labels [45]. Trust can also decrease when there is no clarity about who is behind the certification or how a labelled product is superior to non-labelled products [46].

### 3.4. Selection, Purchase, and Consumption

The effect of sustainability labelling of food products on consumer behaviour in real-world settings is under researched, but in general, evidence thus far suggests an increase in consumer selection and purchase of more sustainable foods. The most recent and relevant systematic review of evidence in this area was published by Potter et al. (2021), who found that 60 out of 76 interventions that were included showed an increase in selection, purchase, or consumption of foods with environmental impact labelling. The paper did not include a meta-analysis of the effect sizes, only an analysis of the effect directions (i.e., supporting the hypothesis, not supporting or mixed results). The review only included studies on environmental sustainability labels. Those examining social responsibility labels, such as Fairtrade, animal welfare, or genetic modification, were excluded. Additionally, the review only included studies with an experimental intervention design and excluded research where participants were not randomized, as well as studies that only used qualitative methods. Most of the included studies were conducted in hypothetical environments, but there was evidence of a significant increase in the selection or purchase effects in studies from real consumer settings as well. Ten studies on actual purchasing behaviour were included, and the labelling interventions showed significant increases in nine of them. The review did not find enough evidence to conclude whether logo-only, text-only, or a combined ecolabel format had a greater impact. There was modest evidence that labelling influenced women, particularly, those of higher income or education, but no conclusive evidence about the role of age was found [4].

Consumers make trade-offs when making food purchasing decisions, and there can be tensions between environmental impact information, price, product perceptions, nutritional quality, and other factors. The habitual nature of supermarket shopping can also reduce the effect of sustainability labels. Food purchasing decisions generally happens with low-involvement processing, meaning that people usually do not put much thought into these decisions. Consumers who buy the same items regularly often overlook competing products, which can reduce the visibility and effect of sustainability labels [21].

Recent studies attempted to understand trade-offs when combining environmental and nutrition labelling on food products, but they have found mixed effects so far [17,47]. Potter et al. (2022b) found a small but significant reduction (−2.0%) in the environmental impact of shopping baskets, compared to the control group (no labelling), when products in a virtual online supermarket had environmental impact and nutrition labelling. However, there was no statistically significant difference in the effect compared to the group that saw only environmental impact labelling (−2.3%) [17]. De Bauw et al. (2022) found significant effects toward lower environmental impact purchases in an online virtual supermarket study among 1000 Belgian household food decision-makers when shown products with a combined Nutri- and Eco-Score. Initially, the combined labelling only improved the nutritional quality of the participants’ baskets, not the environmental impact, but when combined with product recommendations, basket scores of environmental impact, and social norm messages on peers’ eco scores, the sustainability score significantly increased. There was an almost 0.2-point reduction in the environmental impact index used by the researchers, which was approximately four times the effect when only product eco-scores were shown [48]. This suggests that labelling may have a greater impact if used alongside complementary strategies to influence consumer behaviour.

### 3.5. Willingness to Pay

There is evidence that people are willing to pay a premium for food and drink products with environmental, social, or animal welfare labelling. A 2021 systematic review of discrete choice experiments (*n* = 43) in real or virtual settings, testing consumers’ willingness to pay (WTP), concluded that the study participants reported a willingness to pay more and that the effect was stronger for meat and dairy products compared to seafood, nuts, vegetables, and fruits. Female, younger participants, and, in contrast to other sustainability labelling research [4], those from lower educational backgrounds were willing to pay a greater price premium. The review included labelling related to organic certification and any labelling conveying messages relevant to environmental sustainability, but not labelling linked to animal welfare standards. The authors also conducted a meta-analysis on 35 studies with usable data and calculated that participants were willing to pay a premium of 3.79 PPP$/kg (Purchasing Power Parity dollars) for foods with sustainability labelling [5].

Several studies and reviews found that organic labelling leads to the highest reported or documented levels of WTP. The systematic review conducted by Potter et al. (2021) concluded that organic labelling was valued more highly than more specific environmental sustainability labels. Abdu and Mutuku (2021) conducted a meta-analysis of WTP experiments on sustainability labelling on coffee and found organic labelling to be a more significant driver of effects compared to other attributes. The research aimed to estimate the average effect size for each attribute from 97 observations pulled from 22 studies. The weighted average effect size across the studies, in terms of WTP a premium for sustainably labelled coffee, was $1.36/pound. Further analysis revealed that the organic attribute was the most important factor affecting the WTP, having a greater influence than Fairtrade or country-of-origin labelling [49].

Studies found inconsistencies in the role of socio-demographics in WTP for organic products. Katt and Meixner (2020) reviewed studies on WTP for organic food and found conflicting evidence that consumer age, gender, income, education, or household size predicts WTP. However, there was strong evidence that consumer values and attitudes, primarily related to support for environmental concerns and a strong interest in personal health, increased the WTP. These attitudes were assessed in over 30 studies included in the review, and >75% of the observations found a significant effect on WTP [50].

Studies have found evidence of Fairtrade-labelled coffee [51], chocolate [52], and peppers [53] increasing the WTP. But as seen in other research, there is inconsistent evidence of WTP for different consumer segments. Luckstead et al. (2022) used a choice experiment to assess consumers’ willingness to pay a premium for social sustainability labelling on chocolate (“child-labour-free”). Participants in Belgium, France, the UK, and the USA were willing to pay more than a 2.81% price premium, which would be the threshold needed for farmers to eliminate hazardous forms of child labour. Other research points to sustainability labelling with a personal health benefit being linked to an increased WTP, such as pesticide-free food labels [54].

A recent survey among consumers in all 27 EU member states included a willingness to pay discrete choice experiment on animal welfare labelling, which showed that consumers were willing to pay a premium for these products, but with limitations. Consumer preference for lower prices, in general, was higher compared to their willingness to pay for organic foods. In more than two-thirds of the Member States, the majority said they would not pay as much or more for a high-animal-welfare product compared to what they pay for organic products. When people were asked about price premiums for specific components of animal welfare, there was one for which people, on average, were willing to pay even more than for organic products, i.e., assurances that the animals are raised in conditions with outdoor access [27]. A 2016 Eurobarometer survey found similar results, with 59% of the surveyed consumers saying they would be willing to pay for products sourced from animal welfare-friendly production systems. Those results also showed that most EU consumers are unwilling to pay a very high premium for these standards. Among those willing to pay more, most (59%) said they would pay up to a 5% premium. Over a quarter of the respondents (27%) would pay up to 10% more, and only 14% said they would pay a premium of more than 10% [55]. A review of studies on animal welfare standards for fish products from the U.S.A., Europe, and China found similar results, showing that consumers were willing to pay a “moderate” price premium for improved fish welfare or for those that minimize by-catch [56]. These results show that the pricing of animal welfare-labelled products is a key factor in influencing consumer purchasing habits.

## 4. Discussion

This review indicates that there is an appetite for sustainability labelling among consumers. However, few people perceive labelling as the most critical tool in making sustainable choices, signalling the importance of having complementary initiatives to shift consumer behaviour. While the increasing support for sustainability labelling is promising, there are also significant challenges in terms of consumers’ comprehension of these labels. The review underscored several instances of consumer misunderstanding of sustainability information, particularly when provided as specific numerical values such as carbon footprint data. With the growing number of different sustainability labels and logos, it is unclear whether this will increase consumer awareness and lead to behaviour change or instead create more confusion. The review did not find any clear evidence of how consumers manage and interpret this abundant information, which includes not only sustainability information and claims but also other forms of food labelling (e.g., nutrition, health claims, ingredients, origin information, allergens, etc.).

The findings suggest that sustainability labelling can influence consumer attitudes and buying behaviour. The absence of standardised sustainability labelling across food products poses a significant challenge when attempting to draw general conclusions from studies on its effects. Unlike standardised formats found in nutrition labelling and ingredient lists, the voluntary nature of sustainability labelling, coupled with their varying formats, can complicate the generalisation of the research outcomes. Therefore, there is a need for a cautious interpretation of findings from diverse studies.

Evidence shows that consumers are generally willing to pay moderately more for sustainability-labelled products a (e.g., organic labelling often leads to the highest reported willingness to pay). However, there is inconsistency in the role of socio-demographics in determining the willingness to pay. Instead, consumer values and attitudes related to environmental concerns and personal health are more reliable predictors. The review also found that the role of demographics (such as gender and education level) in consumer behaviour towards sustainability labelling in general is not clear, as the results are inconsistent across studies. The role of identity considerations was rarely addressed in the studies included in this review. Given the significance of identity and intersectionality in shaping consumer behaviours and responses to sustainability labelling, future research should explore the complexities of consumer reactions through this lens.

The issue of trust emerged as an important factor affecting the impact of environmental, social, or animal welfare labelling. As consumers become increasingly aware of greenwashing practices, scepticism towards sustainability information and claims may rise. There is a marked preference for labelling backed by governments or public authorities, as opposed to industry-driven initiatives. However, this trust varies regionally and may be influenced by cultural norms and institutional trust levels.

Considering these findings, it is essential to explore optimal ways to present sustainability information and claims to consumers. Simplicity, accuracy, and intuitively understandable cues are crucial factors for labelling to influence consumer behaviour. The use of visual cues, such as traffic light colours, may also aid consumer understanding. Composite labels that synthesise nutrition and sustainability information appear to be a promising approach, provided they convey information simply and understandably.

The primary limitation of this review is that the literature search did not follow a formal systematic review protocol. However, given the broad scope of the research question, a narrative review was felt to be a more practical option to understand the current state of knowledge and identify gaps in the research. The review also built upon a systematic review on environmental sustainability labelling that members of the team were involved in [4], including using and expanding on its keyword search strategy to identify a wide range of potentially relevant papers. While the research team further expanded the scope by exploring grey literature, including a broad and inclusive questionnaire sent to European label owners and administrators, the questionnaire suffered from a low response rate.

To build stronger evidence in this field, future research should focus on real-world settings, such as supermarkets, and include more representative populations to generate transferable insights. Longitudinal data examining consumer behaviour over time would also provide valuable information. Additionally, attention should be given to different demographic groups to understand which presentations have a greater impact on those who are sceptical of claims, value sustainability concerns less, or are more price-sensitive. Exploring the role of the messenger in labelling schemes is crucial, as government or NGO-backed labelling may foster greater trust among consumers.

Sustainability labelling has the potential to be a useful tool among a suite of interventions to encourage sustainable food choices. However, the impact of labelling schemes on actual consumer behaviour remains limited, with an intention–action gap often observed. Future research should investigate the most successful ways to present sustainability information to consumers and address the influence of demographic factors, values, and attitudes on consumer behaviour.

## Figures and Tables

**Figure A1:**
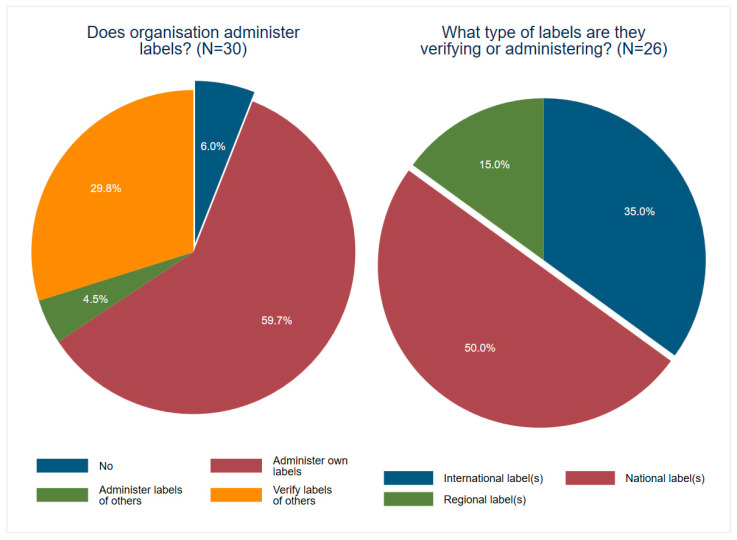
Relationship to Sustainability Labelling and Types.

**Figure A2:**
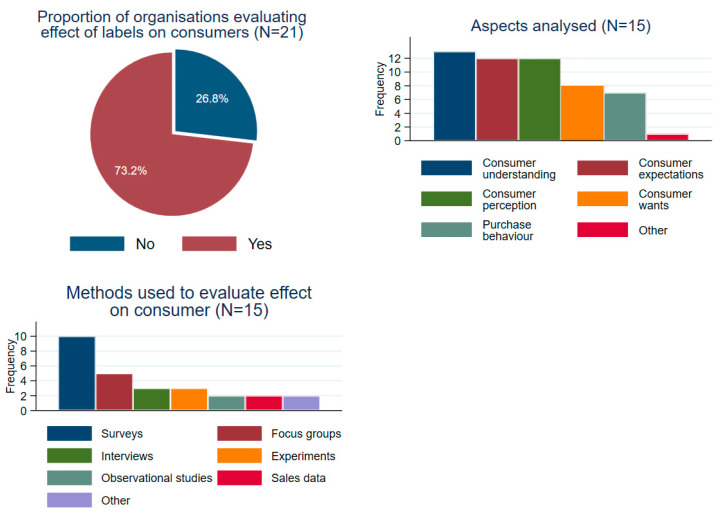
Number and Types of Labelling Evaluations Conducted.

**Figure A3:**
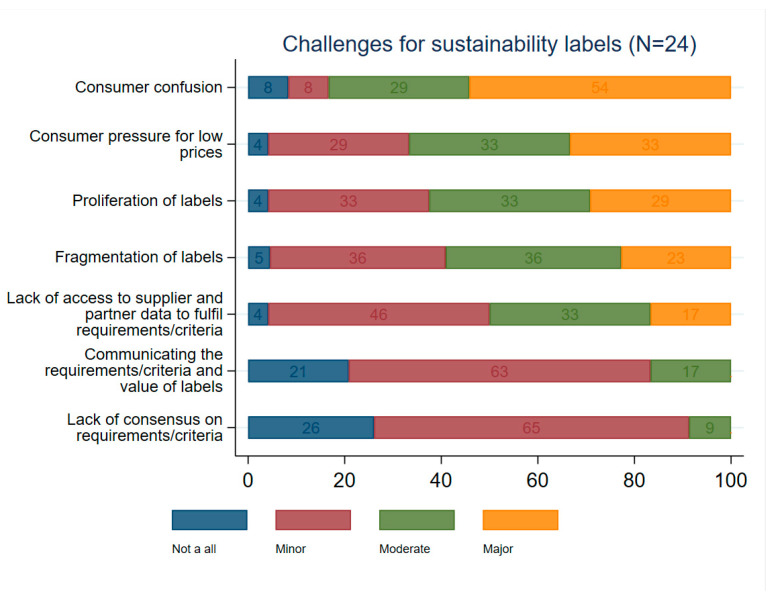
Perceived Challenges of Sustainability Labelling.

## Data Availability

Data sharing is not applicable to the literature review as no datasets were generated. However, the data presented in this article related to the label owner survey are available on request from the corresponding author.

## Appendix A. Analysis of Label Owner Questionnaire

Between August and September 2022, the European Commission’s Joint Research Centre conducted an online questionnaire targeting European sustainability-related label owners and third-party verification providers, identified in a previous mapping study. The questionnaire aimed to gather information about the types of sustainability food labelling that organisations administer or manage and whether any evaluation had been conducted on their effects on consumer attitudes or behaviour. Feedback was also requested about perceived challenges in implementing sustainability labelling on food and drink products. The questionnaire was distributed to 192 organizations and received 30 completed questionnaires (a response rate of 15.6%). Seven of the fifteen respondents (46.7%) who reported having evaluated labelling shared the related documents, which were included, where relevant, in this review.
